# The metabolism-related lncRNA signature predicts the prognosis of breast cancer patients

**DOI:** 10.1038/s41598-024-53716-7

**Published:** 2024-02-12

**Authors:** Xin Ge, Shu Lei, Panliang Wang, Wenkang Wang, Wendong Wang

**Affiliations:** 1 Department of Breast Surgery, The First Affiliated Hospital of Zhengzhou University, 1 Jianshe East Road, Erqi District, Zhengzhou, 450052 China; 2 Department of Gynecology and Obstetrics, The Third Affiliated Hospital of Zhengzhou University, No.3 Kangfu Middle Street, Erqi District, Zhengzhou, 450052 China

**Keywords:** Breast cancer, Bioinformatics, Metabolism-related lncRNAs, Risk score, Prediction signature, Cancer, Genetics, Biomarkers, Risk factors

## Abstract

Long non-coding RNAs (lncRNAs) involved in metabolism are recognized as significant factors in breast cancer (BC) progression. We constructed a novel prognostic signature for BC using metabolism-related lncRNAs and investigated their underlying mechanisms. The training and validation cohorts were established from BC patients acquired from two public sources: The Cancer Genome Atlas (TCGA) and Gene Expression Omnibus (GEO). The prognostic signature of metabolism-related lncRNAs was constructed using the least absolute shrinkage and selection operator (LASSO) cox regression analysis. We developed and validated a new prognostic risk model for BC using the signature of metabolism-related lncRNAs (SIRLNT, SIAH2-AS1, MIR205HG, USP30-AS1, MIR200CHG, TFAP2A-AS1, AP005131.2, AL031316.1, C6orf99). The risk score obtained from this signature was proven to be an independent prognostic factor for BC patients, resulting in a poor overall survival (OS) for individuals in the high-risk group. The area under the curve (AUC) for OS at three and five years were 0.67 and 0.65 in the TCGA cohort, and 0.697 and 0.68 in the GEO validation cohort, respectively. The prognostic signature demonstrated a robust association with the immunological state of BC patients. Conventional chemotherapeutics, such as docetaxel and paclitaxel, showed greater efficacy in BC patients classified as high-risk. A nomogram with a c-index of 0.764 was developed to forecast the survival time of BC patients, considering their risk score and age. The silencing of C6orf99 markedly decreased the proliferation, migration, and invasion capacities in MCF-7 cells. Our study identified a signature of metabolism-related lncRNAs that predicts outcomes in BC patients and could assist in tailoring personalized prevention and treatment plans.

## Introduction

Breast cancer (BC), a common malignancy in women, is the most common cancer and the second most common cause of cancer-related deaths globally^1,2^. The molecular mechanisms driving BC pathogenesis have been extensively researched and categorized into three major subtypes (luminal, HER2-enriched, and triple-negative breast cancer) based on markers such as expression of estrogen receptor (ER) and progesterone receptor (PR)^3^. However, a robust molecular signature is still needed to accurately predict and stratify disease outcomes^4^. Dysregulated metabolism is a prominent feature of BC^5^. Significant focus has been directed towards analyzing the metabolic alterations that accompany the onset and progression of cancer^6^. The Warburg effect is a well-known phenomenon in which tumor cells transition from oxidative phosphorylation to glycolysis for energy generation^7^. The metabolic changes in cancer cells facilitate the production of ATP as well as the necessary metabolic intermediates needed for cellular growth and proliferation, including amino acids, fatty acids, and nucleotides. High-throughput analytical methods have unveiled the metabolic alterations linked to the mechanisms of BC development^8,9^. Research on metabolism has provided insights into novel therapeutic approaches and drug development^10^. Variations in metabolic activity among tumor cells in different patients necessitate the identification of metabolism-related biomarkers, which can reveal the molecular mechanisms of tumor progression, ultimately enhancing the development of effective treatment strategies and improving prognoses for BC patients^11^. Dai et al. developed a prognostic model for BC based on long noncoding RNAs (lncRNAs) associated with amino acid metabolism^12^. Xu et al. found that glucose metabolism-related lncRNAs could impact the progression of BC^13^. Shi et al. identified that lipid metabolism lncRNAs held significant prognostic value in predicting the survival of patients with BC^14^. The role of metabolism-related lncRNAs in the progression of BC is still uncertain.

In this study, we aimed to identify a signature of metabolism-related lncRNAs and assess its prognostic value in BC patients. We screened the Cancer Genome Atlas (TCGA) transcriptome data of BC patients to identify clinically significant metabolism-related lncRNAs. Using these lncRNAs, we constructed a prognostic model and externally validated its accuracy in a GEO dataset. We also investigated immune infiltration, immunotherapy, and medication sensitivity in high- and low-risk groups. A unique model of metabolic risk score was developed to predict the prognosis and therapeutic responsiveness of BC patients. Furthermore, we validated specific risk factors, such as C6orf99, in BC cell lines.

## Methods and materials

### Data extraction

We obtained the transcriptomic datasets and clinical information from the TCGA databases (https://portal.gdc.cancer.gov/) and the Gene Expression Omnibus (GEO dataset GSE58644, based on the GPL6244 platform, https://www.ncbi.nlm.nih.gov/geo). After excluding cases with missing clinicopathological information, only patients diagnosed with BC and with an overall survival exceeding 30 days were considered for the analysis. The study utilized RNA sequencing data from the TCGA database, comprising 1022 BC and 112 adjacent non-tumor cases, along with 312 BC from the GSE58644 dataset.

### Metabolism‐related lncRNAs detection

The R package was used to identify differentially expressed genes associated with metabolism in BC and normal tissues^15^. A total of 944 genes related to metabolism were identified using the Molecular Signatures Database (http://www.broad.mit.edu/gsea/msigdb/) and the Kyoto Encyclopedia of Genes and Genomes (KEGG) gene sets. Subsequently, the expression levels of metabolism-related genes and their corresponding lncRNAs were compared using Pearson correlation coefficients. To identify metabolism-related lncRNAs, the correlation coefficient and p values were used (|Cor _pearson_|> 0.4 and *p* value < 0.001), we screened 1135 metabolism-related lncRNAs that met these criteria.

### Construction of a risk signature

Metabolism-related lncRNAs showing differential expression between tumor and normal tissues in the TCGA cohort were identified using the limma package, applying fold change (FC) > 2 and a false discovery rate (FDR) < 0.05 as cut-off criteria. Univariate Cox proportional hazards regression analysis was used to identify metabolism-associated lncRNAs significantly linked to the prognosis of BC. Subsequently, multivariate Cox regression analysis was applied to pinpoint metabolism-related lncRNAs for the development of a predictive signature. Protective factors exhibit a hazard ratio (HR) of 1 or less, whereas risk factors demonstrate a HR greater than 1. The computational formula was constructed for this analysis as follows: Risk score = ${\sum }_{i=1}^{n}(Expi* Coei)$. The number of prognostic genes was denoted as *n*, the expression of metabolism-related lncRNAs was denoted as *Expi*, and the regression coefficient of the metabolism-related lncRNA in the model was denoted as *Coei*. Patients in the two cohorts were stratified into low-risk or high-risk groups based on the median value of their risk scores in the training cohort. To evaluate the prognostic efficacy of the risk score model, we employed receiver operating characteristic (ROC) curve analysis and principal component analysis (PCA) to visualize lncRNA expression patterns in the two groups of BC patients.

### Prognostic signature evaluation

Cytoscape was used to display and visualize correlations between mRNA and lncRNA co-expression, while the corrplot software was employed to construct interactions between identified lncRNAs. The co-expressed network components were represented by a sankey diagram in the R package. The biological functions and pathways associated with the identified lncRNAs were explored using gene ontology (GO) and KEGG pathway analysis. Immune signatures, associated markers, and estimated gene sets for immune scores were utilized to infer immune infiltration through single-sample gene set enrichment analysis (ssGSEA)^16^. The CIBERSORT algorithm was utilized to assess the proportion of tumor-infiltrating immune cells in both groups^17^. The Tumor Immune Dysfunction and Exclusion (TIDE) algorithms (available at http://tide.dfci.harvard.edu/) were employed to forecast the clinical responses to immune checkpoint inhibitors^18^. R package “pRRophetic” was used to assess the effectiveness of chemotherapy drugs by half maximal inhibitory concentration (IC50) of each BC patient^19^.

### Nomogram construction

The rms package was utilized to conduct multivariate Cox regression analyses with a risk score model in the training cohort, which were then integrated with clinicopathological features to construct a nomogram. The integration of these prognostic indicators, along with the computation of the concordance index (C-index) and calibration curves, was utilized in the development of a nomogram for predicting one-, three-, and five-year OS probabilities.

### Cell culture

The human normal breast cell line (MCF-10A) and BC cell lines (MCF-7, T47D, MDA-MB-231, and HCC1937) were sourced from the National Infrastructure of Cell Line Resource in Beijing, China. They were maintained in RPMI‐1640 medium (HyClone) supplemented with 10% fetal bovine serum (Gibco) at 37 °C in a 5% CO_2_ incubator.

### qRT-PCR and transfection

TRIzol (Invitrogen) was used to extract total RNA from the cell line. The FastKing RT Kit (TIANGEN Biotech, Beijing, China) was employed to synthesize cDNA following the manufacturer's protocol. The FastKing One Step Kit (TIANGEN Biotech, Beijing, China) was utilized to perform qRT-PCR following the manufacturer's protocol. Relative expression level of C6orf99 was calculated by the 2^−∆∆Ct^ method. C6orf99 specific targeting siRNA (si-C6orf99 #1 and si-C6orf99 #2) and negative control siRNA (siNC) were purchased from Sangon Biotech. The siRNA was transfected using Lipo2000 (Invitrogen) according to the manufacturer’s protocol. The primers and siRNA were described in Supplementary Table 1.

## CCK-8, and Transwell assay

The cell counting kit 8 (CCK-8, KeyGEN BioTECH) was utilized to quantify cell proliferation. 2000 cells were plated in each well using 96-well plate and CCK-8 reagent was added to each well. Then, the plates were incubated at 37 °C for 1–2 h. Absorbance value at a wavelength of 450 nm were utilized to quantify the cell number. Cell migration and invasion were measured by Transwell insert (NEST). The Transwell insert was coated with Matrigel for detecting cell invasion ability or without Matrigel for detecting cell migration ability. 60,000 cells in serum‐free medium were added into the upper of a Transwell insert and the lower chamber filled with medium with 20% FBS for 1–2 days.

### Statistical analyses

Statistical analyses were performed using R (version 4.0.2). A Pearson correlation coefficient was calculated for further analysis. The Kruskal–Wallis and Wilcoxon tests were employed to assess the expression of DEGs in normal and malignant tissues, respectively. The univariate Cox regression model was utilized to calculate the HR and corresponding 95% confidence intervals (CIs). The coefficients of the prognostic signatures were determined using the absolute shrinkage and selection operator (LASSO) regression. Survival curves were generated using the Kaplan–Meier method. We utilized the log-rank test to compare OS and RFS between groups. Cox proportional hazard models, both univariate and multivariate, were utilized to examine independent risk variables for the prognosis of BC patients. *P*-value < 0.05 was regarded as indicative of a significant difference in the statistical analyses.

## Results

### Metabolism‐related lncRNA identification in the TCGA cohort

**Figure 1:**
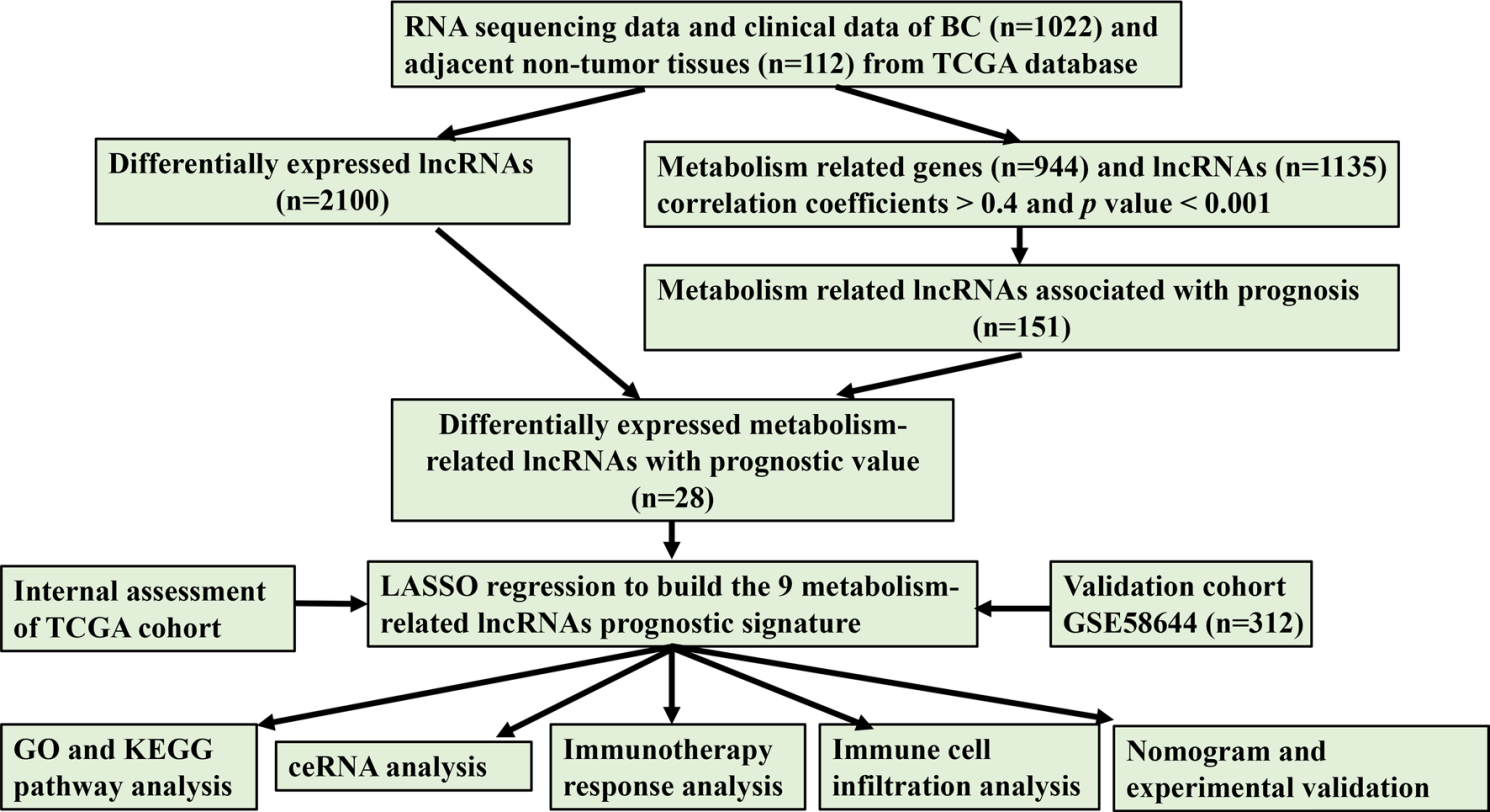
The flowchart of our research.

**Figure 2:**
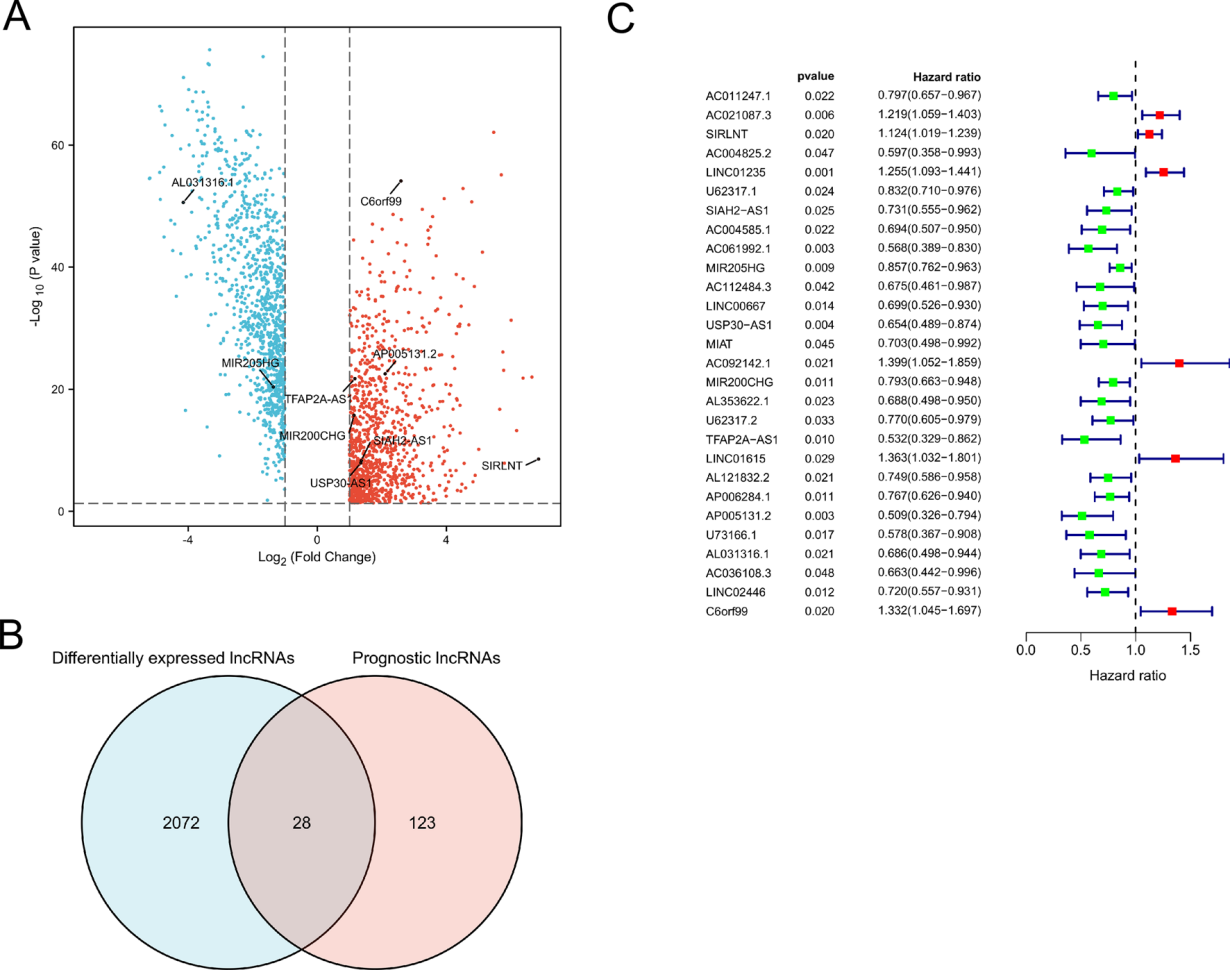
Exploration of metabolism‐related lncRNAs in BC. (**A**) lncRNA expressed differently in tumor and normal tissues. Up-regulated lncRNAs were shown in red, while down-regulated lncRNAs were shown in blue. (**B**) Venn diagram showing lncRNAs met two criteria. 9 lncRNAs were tagged in (**A**). (**C**) After further filtering, the metabolism‐related lncRNAs that were substantially linked with prognosis.

The flowchart of the study was exhibited in Fig. 1. To identify metabolism-related lncRNAs in the TCGA cohort, we analyzed a dataset consisting of 14,142 lncRNAs and 19,658 mRNAs. Among these, 2100 lncRNAs exhibited differential expression between patient tumors and normal tissues (Fig. 2A). We retrieved 944 genes associated with metabolism from the KEGG pathway database and screened for significant metabolism-related lncRNAs through significant univariate Cox regression analysis. This analysis identified 151 lncRNAs significantly correlated with BC survival and further investigations considered 28 metabolism-related lncRNAs as candidates based on their differential expression and prognostic significance (Fig. 2B,C).

### Construction of a risk signature for prognostic

After identifying metabolic lncRNAs associated with candidate prognosis, we used LASSO regression models to construct the prognostic signature, incorporating the expression of 9 lncRNAs (SIRLNT, SIAH2-AS1, MIR205HG, USP30-AS1, MIR200CHG, TFAP2A-AS1, AP005131.2, AL031316.1, and C6orf99). Each coefficient in the signature represents the expression weight of the corresponding lncRNA. By combining the expression levels of these 9 metabolism-related long noncoding RNAs with their associated Cox regression coefficients, we generated a risk score for each BC patient (*P* < 0.05, Table 1).

**Table 1:** Prediction signature for survival.

lncRNA	Coefficient
SIRLNT	0.097448759
SIAH2-AS1	− 0.332363482
MIR205HG	− 0.098220261
USP30-AS1	− 0.416509153
MIR200CHG	− 0.13935231
TFAP2A-AS1	− 0.666605613
AP005131.2	− 0.478339835
AL031316.1	− 0.221521122
C6orf99	0.222236591

### Establishing and validating a robust metabolism-related lncRNAs prognostic signature

**Figure 3:**
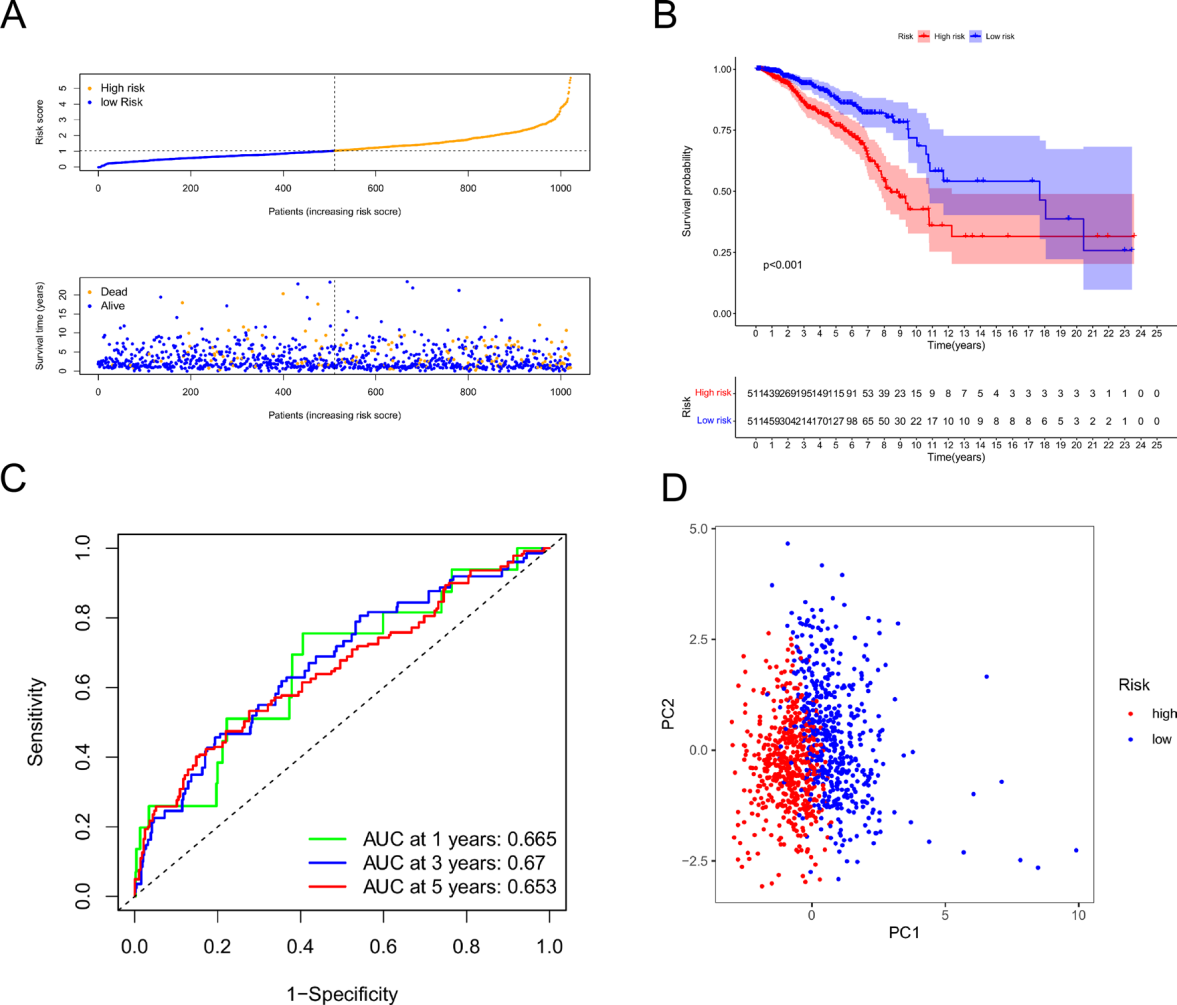
Signature test in the training cohort. (**A**) Risk score and survival status distribution of BC patients in low-risk and high-risk groups. (**B**) OS survival curves for low-risk and high-risk patients. (**C**) Risk score ROC Curve for one, three, and five years. (**D**) PCA visualization of risk categorization.

**Figure 4:**
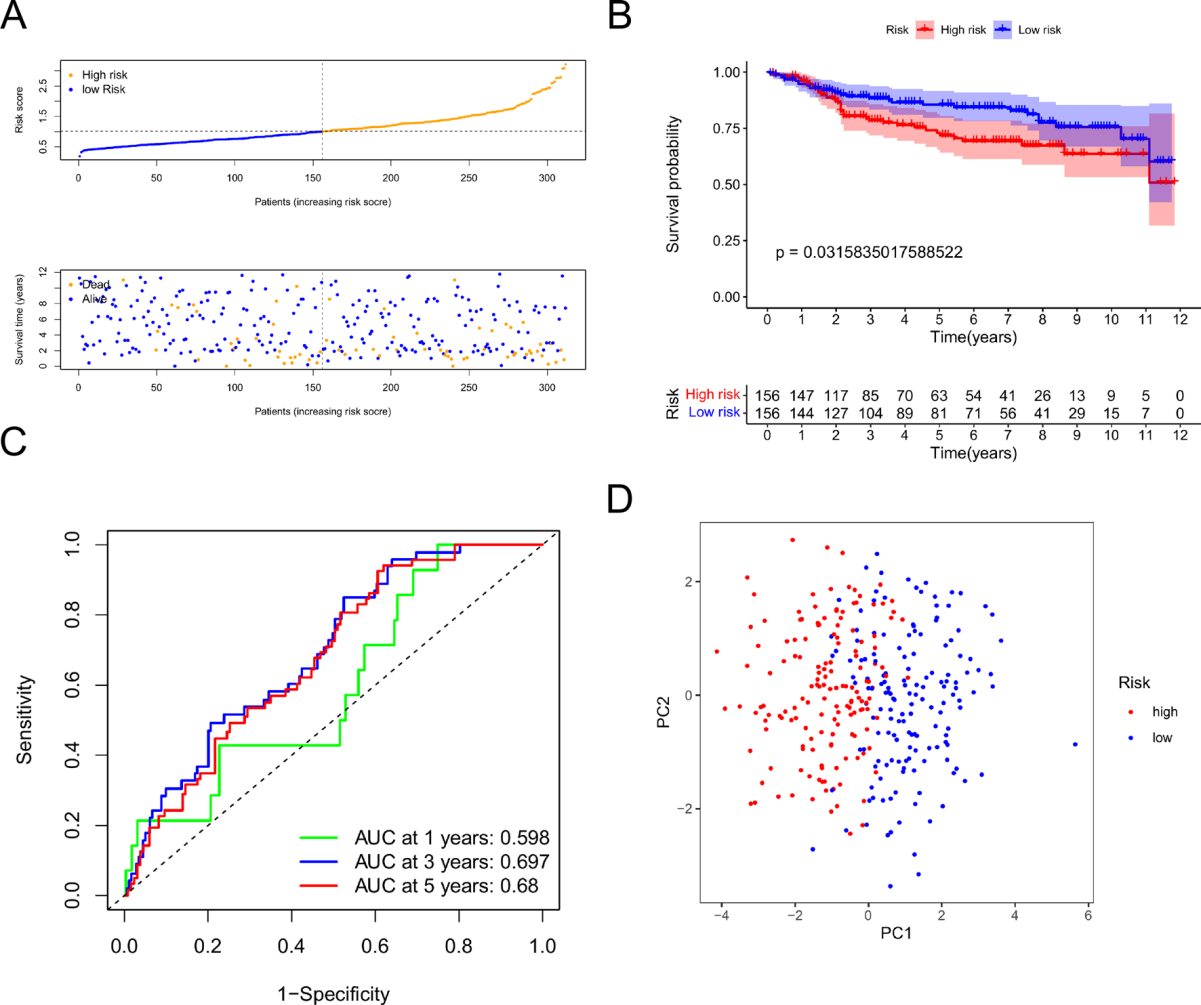
Signature test in the validation cohort. (**A**) Risk score and survival status distribution of BC patients in low-risk and high-risk groups. (**B**) OS survival curves for low-risk and high-risk patients. (**C**) Risk score ROC Curve for one, three, and five years. (**D**) PCA visualization of risk categorization.

To establish the robustness of our metabolism-related lncRNAs prognostic signature, we calculated the risk score in the TCGA cohort for internal validation and in the GEO cohort for external confirmation. Based on their median risk scores, the 1022 BC patients from TCGA and the 312 BC patients from GEO were classified into high- and low-risk categories. The high-risk groups in both the training cohort (91/511 vs. 49/511) and the validation cohort (41/156 vs. 28/156) exhibited higher mortality rates compared to the low-risk groups (Figs. 3A, 4A). Kaplan–Meier curve analysis demonstrated a significantly lower overall survival rate for high-risk patients than for low-risk patients in both cohorts (Figs. 3B, 4B). The prognostic model showed high predictive power, as indicated by the area under the receiver operating characteristic curve values for predicting 3-year survival in the training (0.67) and validation (0.697) groups (Figs. 3C, 4C). Additionally, we used PCA to examine the distinct distribution patterns of the high- and low-risk groups. The risk model successfully separated breast cancer patients into two groups with different risk levels (Figs. 3D, 4D).

### Constructing the co‐expression network in prognostic model

**Figure 5:**
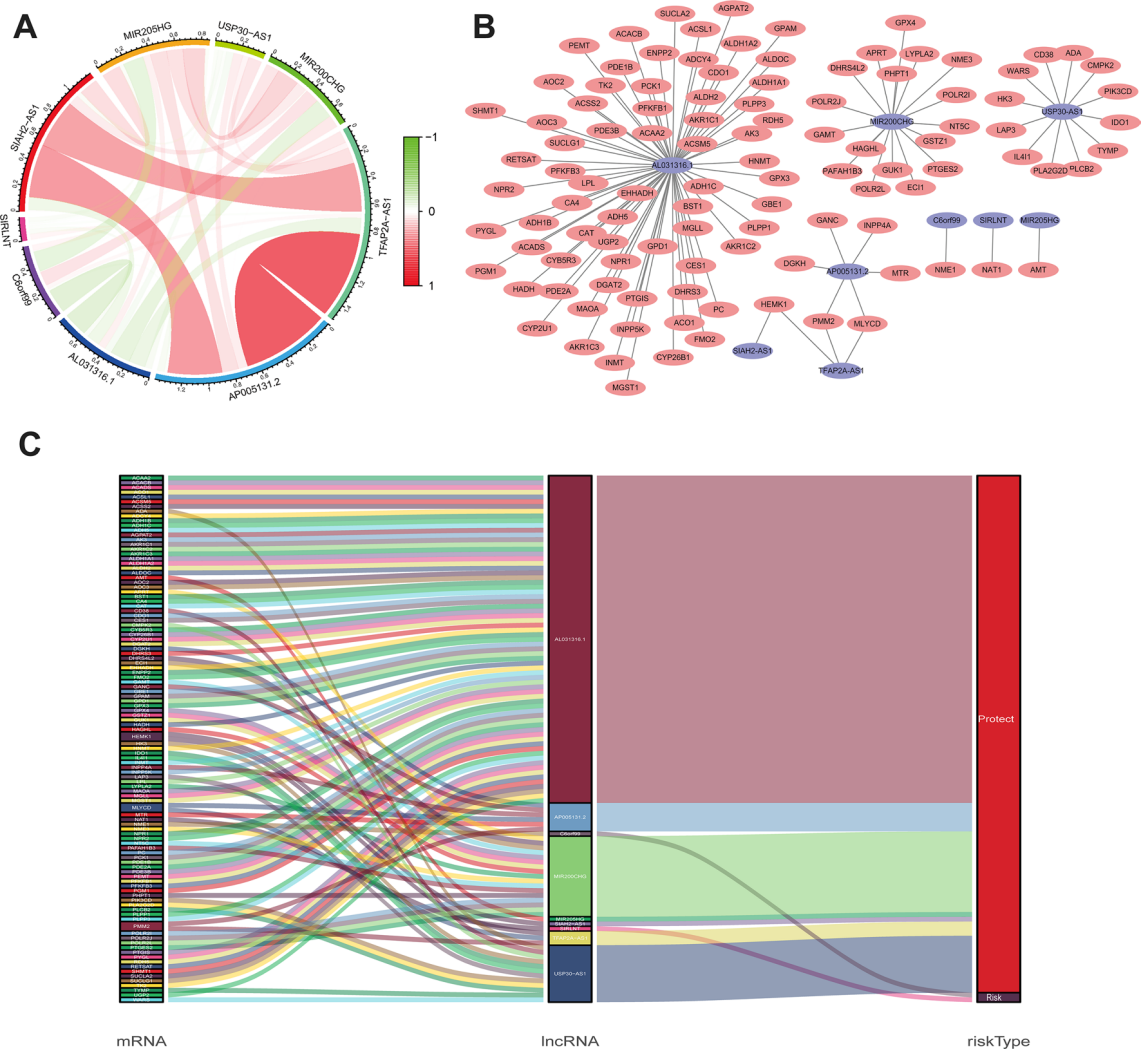
Co-expressed lncrna mRNA of the prognostic signature. (**A**) Annotated coefficients for 9 lncRNAs. (**B**) A metabolic-related lncRNA-mRNA co-expression regulation network. (**C**) Sankey diagram depicting the relationships between mRNAs, lncRNAs, and risk types.

As illustrated in Fig. 5A, the metabolism-related lncRNAs in the prognostic model were highly correlated, which demonstrated the potential consistency of lncRNAs function in the model. In the regulatory mechanisms of metabolism‐related lncRNAs, it is considered that lncRNAs regulate mRNAs in breast cancer onset and development. Cytoscape was used to create a network of co-expressions. In our prognostic signature, there were 111 lncRNA-mRNA couples in the lncRNA-mRNA co-expression network, and 108 mRNAs were substantially linked to metabolism-related lncRNAs (Fig. 5B). AL031316.1, MIR200CHG, and USP30-AS1 were likely to be the most important components. The Sankey diagram established a link between lncRNAs and mRNAs and revealed a link between metabolism-related lncRNAs and overall survival in BC patients (Fig. 5C). Notably, C6orf99 and SIRLNT were the risky factors among the included lncRNAs.

### Discovery of functional enrichment analysis

**Figure 6:**
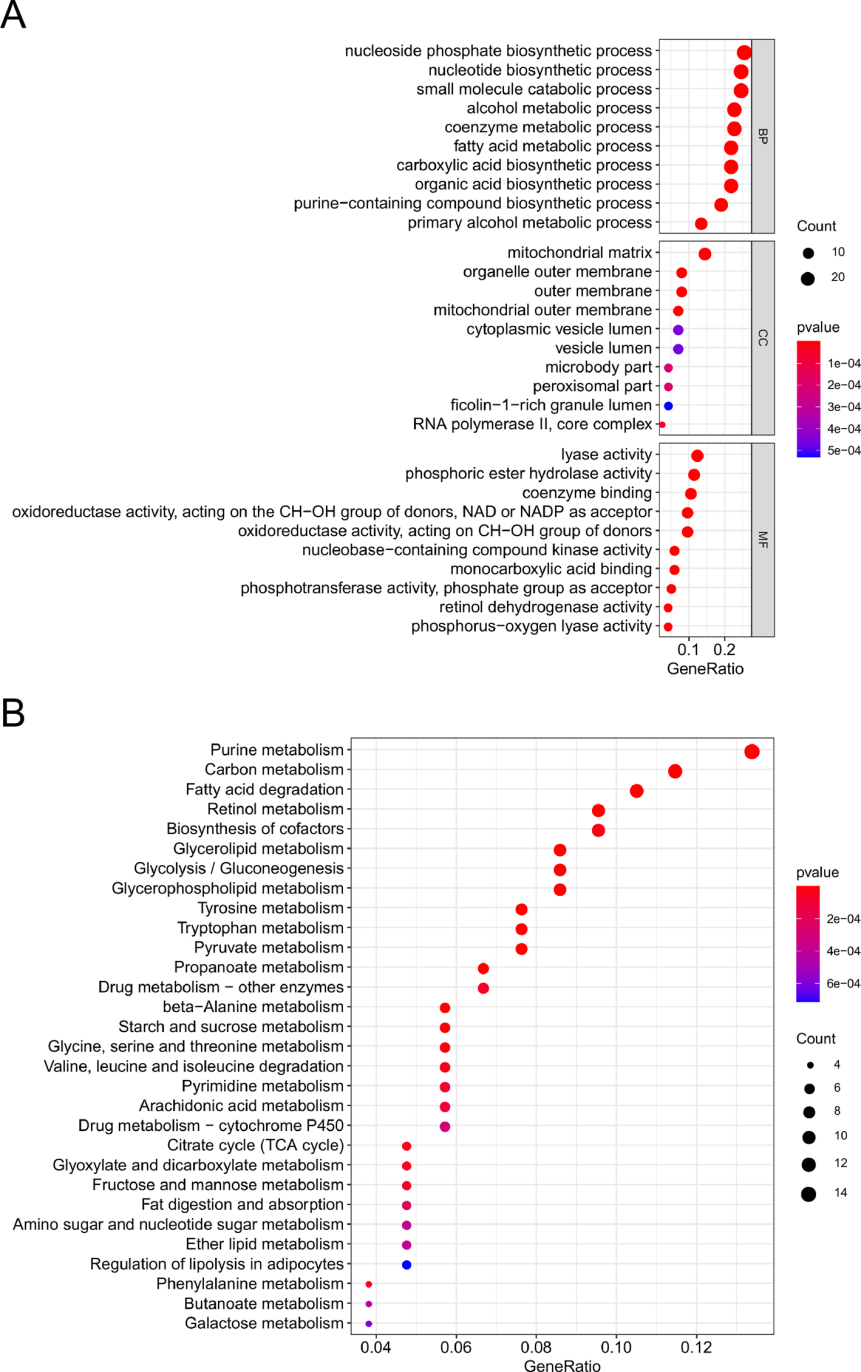
Functional analysis of lncrnas mRNAs co-expression. (**A**) GO enrichment analysis. (**B**) KEGG pathway analysis^52^.

In order to investigate the variations in gene functions and gene enrichment between high-risk and low-risk groups based on the risk model, a total of 111 co-expressed mRNAs were identified. We performed GO analysis of these mRNAs and discovered that the top three biological processes represented by GO terms were the nucleoside phosphate biosynthetic process, nucleotide biosynthetic process, and small molecule catabolic process (Fig. 6A). As expected, KEGG pathway analysis confirmed that these genes were associated with metabolic functions, and the most significantly enriched pathways were those for fatty acid degradation, purine metabolism, and carbon metabolism (Fig. 6B).

### Comparing tumor-infiltrating immune cells in two groups

**Figure 7:**
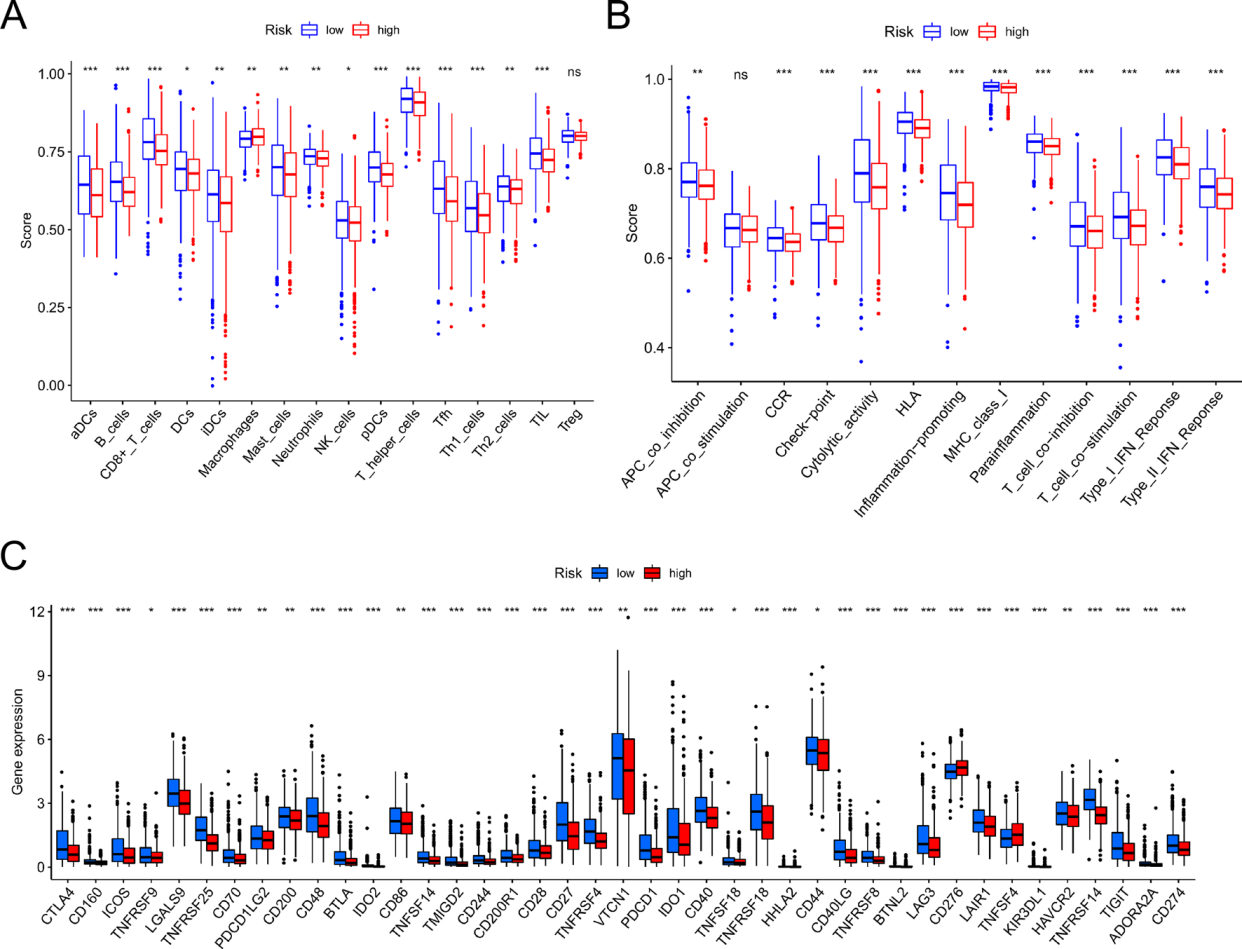
Immune infiltration signature in two groups. (**A**) 16 immune cells in low and high-risk groups. (**B**) 13 immune functions in two groups. (**C**) Known immune checkpoints. *P < 0.05, **P < 0.01, ***P < 0.001.

Immunotherapy is a novel therapeutic option for BC that may enhance antitumor capacity by stimulating patients' immune systems. Nonetheless, not all BC patients are candidates for immunotherapy, implying that identifying these individuals is critical. The immune infiltration of the two risk groups was compared using the TIMER method. The higher the score, the more robust the immunological activity. The immunological activity of innate immunity cells (aDCs, DCs, iDCs, mast cells, and pDCs) and adaptive immune cells (B cells, CD8^+^ T, T helper, Tfh, and until cells) was greater in the low-risk subgroup as compared to the high-risk subgroup (Fig. 7A). Similar results for immune activities such as checkpoint, cytolytic activity, type I IFN response, and type II IFN response were verified using the ssGSEA method (Fig. 7B). Following that, we examined the association between risk scores and important immunological checkpoints. In comparison to the low-risk group, the high-risk group had considerably lower levels of expression of many immunological checkpoints (CTLA4, CD274, and PDCD1) (Fig. 7C). The study revealed that a risk score could be used to help find people who might benefit from immunotherapy.

### Investigation of clinical treatment in risk groups

**Figure 8:**
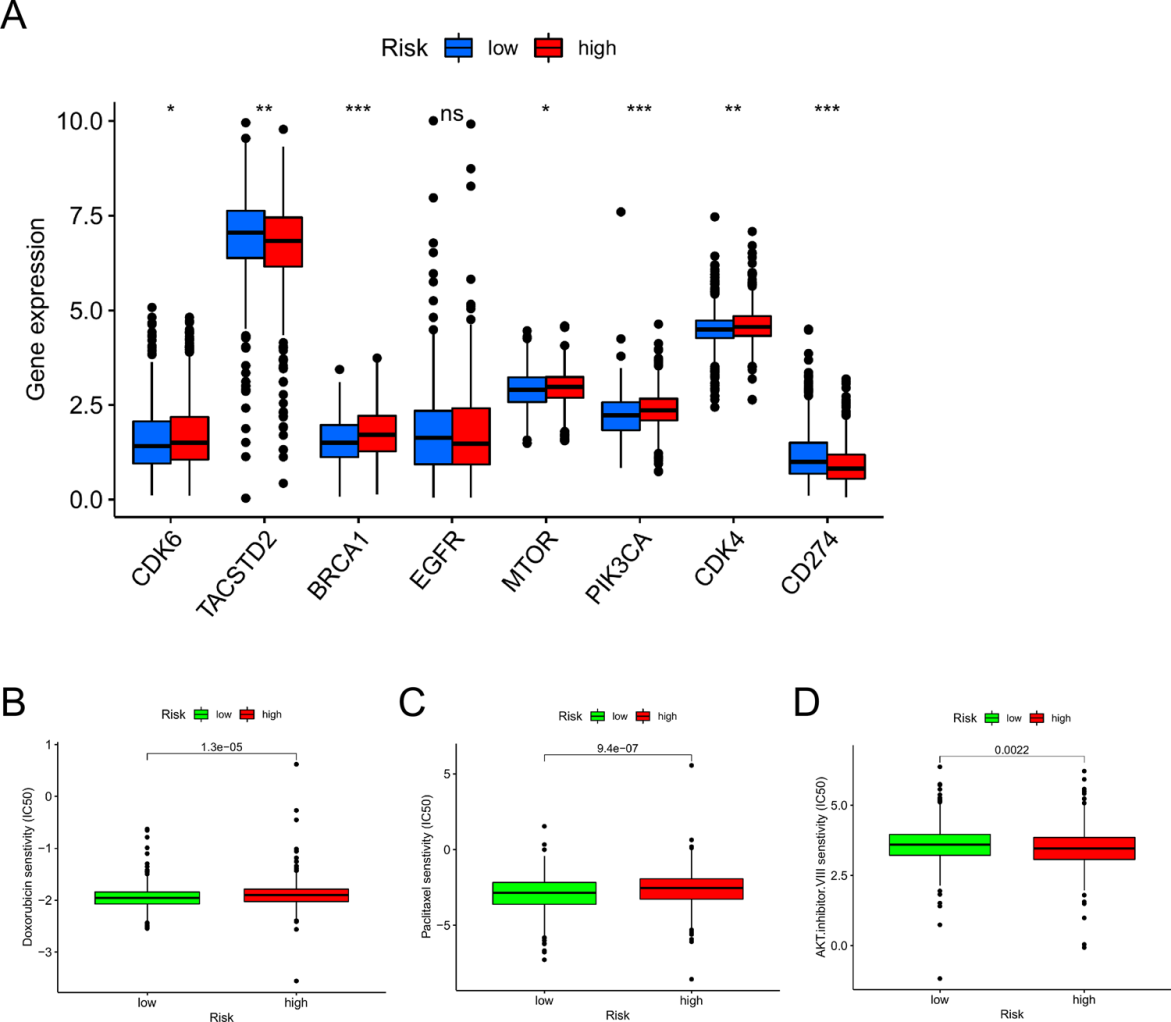
Potential therapeutic targets and drugs for different risk groups. (**A**) Expression of important known drug targets in breast cancer in different risk subgroups. (**B**) The sensitivity to Docetaxel, Paclitaxel, and AKT inhibitor VIII of BC patients. *P < 0.05, **P < 0.01, ***P < 0.001.

Recently, there has been a surge in the development of new molecular target drugs and regimens that are tailored to the predicted sensitivity of specific histological types of BC. Unique biological markers in individual patients can provide tailored therapy, leading to optimal treatment efficacy. The expression of CDK4, BRCA1, PIK3CA, and CDK6 was higher in the high-risk group (Fig. 8A). This allowed us to choose relevant drugs for BC patients based on their risk mode. Additionally, we discovered that docetaxel and paclitaxel, which are used in the treatment of BC, had a greater IC50 in the high-risk group (Fig. 8B). As expected, the high-risk group showed a lower IC50 for AKT inhibitor VIII (Fig. 8B). Based on these findings, individuals in high- and low-risk groups were able to develop tailored treatment plans.

### Evaluation of prognostic value and construction of nomogram

**Figure 9:**
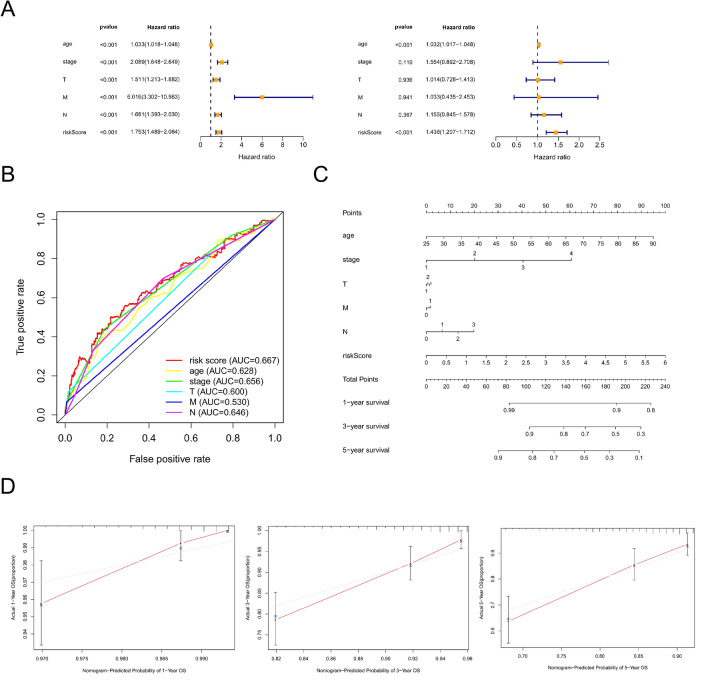
Evaluating risk features and constructing a prognostic nomogram. (**A**) Univariate and multivariate analysis in BC. (**B**) ROC curves of risk model score and clinical features. (**C**) The prognostic nomogram utilized the risk score and clinicopathological characteristics to predict one-, three-, and five-year survival rates. (**D**) Calibration curves demonstrated the concordance between predicted and observed 1-, 3-, and 5-years survival rates based on the nomogram.

The metabolic-related lncRNA prognostic signature was tested using Cox regression analysis to see if it was an independent prognostic factor for BC patients in the TCGA. A univariate Cox regression study found that age, stage, T stage, M stage, and N stage, as well as risk scores, were all significantly linked with overall survival in BC patients, and a multivariate analysis suggested that age and risk scores may be independent predictors of BC survival (*P* < 0.05, Fig. 9A). Furthermore, the prognostic accuracy of the metabolism-related lncRNAs was assessed using a time-dependent receiver operating characteristic (ROC) analysis, with an AUC value of 0.667 (Fig. 9B). The nomogram's C-index value was 0.764. Nomograms are extensively used to calculate a score based on the values of numerous prognostic indicators to estimate patient survival^20^. In patients with BC, this nomogram was used to predict survival rates at 1, 3, and 5 years (Fig. 9C). The calibration curves showed good agreement between expected and actual OS rates after one, three, and five years of follow-up (Fig. 9D). We may conclude from these data that our prognostic nomogram is both accurate and robust.

### Knockdown C6orf99 inhibited MCF-7 cell proliferation, migration, and invasion

**Figure 10:**
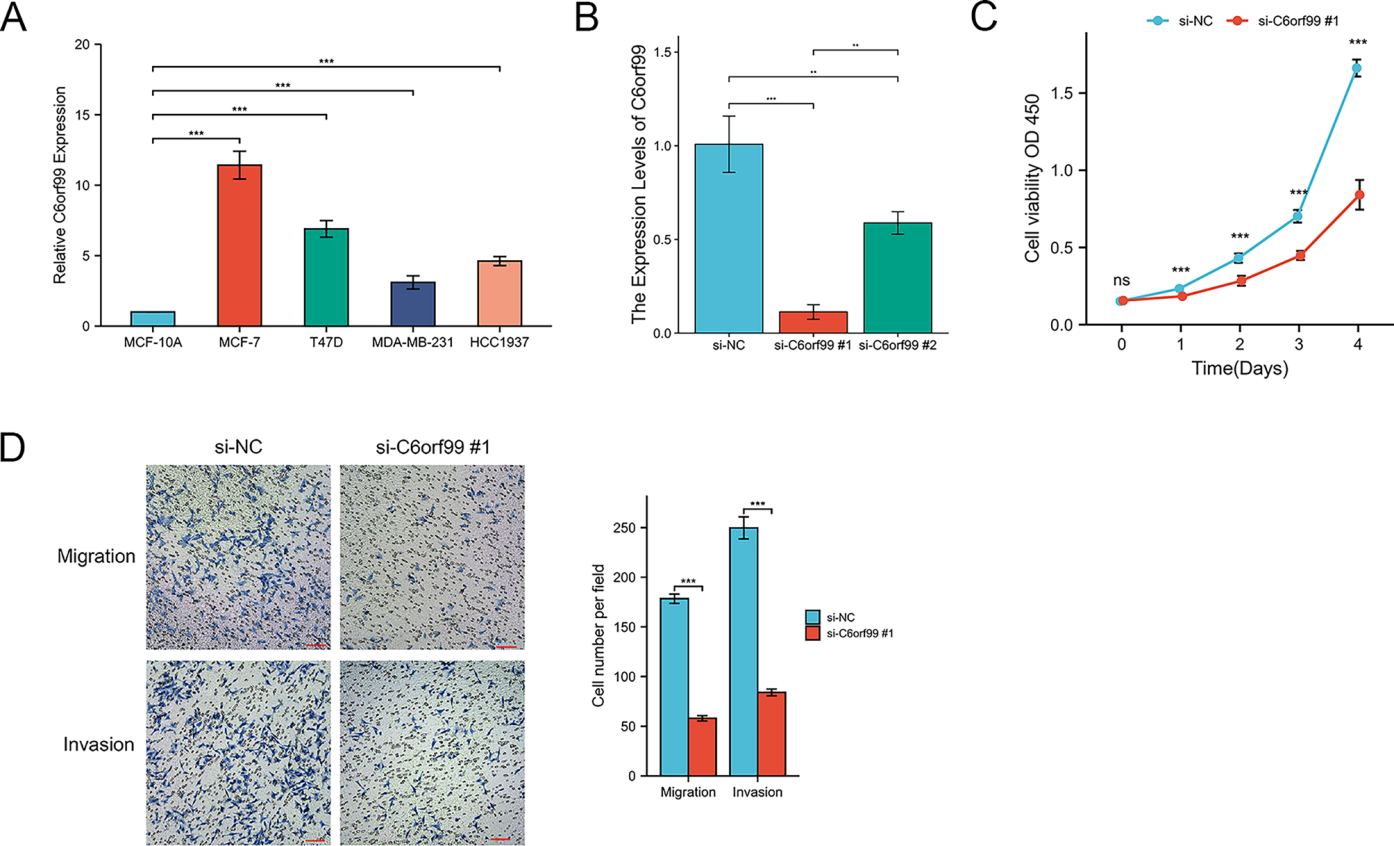
The effects of C6orf99 on BC cell proliferation, migration, and invasion. (**A**) The expression level of C6orf99 in normal and BC cell lines. (**B**) Transfection siRNA efficiency was detected in MCF-7. (**C**) CCK-8 assays were evaluated cell viability in MCF-7. (**D**) Cell migration and invasion were detected by transwell assays in MCF-7. *P < 0.05, **P < 0.01, ***P < 0.001.

In order to investigate the biological function of these lncRNAs, we conducted cytology and molecular biology experiments. Given the number of lncRNAs in the signature, C6orf99 was a risky factor, a new lncRNA which has never been researched. The expression of C6orf99 was significantly higher expressed in BC cell lines (Fig. 10A). Thus, we chose the MCF-7 cell line for molecular validation. We used siRNA to knockdown C6orf99 in MCF-7 and found that si-C6orf99 #1 significantly decreased the expression of C6orf99 (Fig. 10B). The CCK-8 results showed that knockdown of C6orf99 inhibits cell proliferation ability (Fig. 10C). Moreover, knockdown of C6orf99 suppressed migration and invasion in MCF-7 (Fig. 10D). Collectively, these findings indicated that C6orf99 promoted cell proliferation and metastasis in BC cells.

## Discussion

Existing evaluation models for predicting BC prognosis heavily rely on clinical factors, simplifying the process of collection and assessment of patient data^21,22^. However, the AJCC TNM staging primarily employs anatomical data, and it often fails to precisely forecast cancer recurrence^23^. The enhancement of gene detection technologies proposes an alternative approach, enabling hospitals and specialized labs to sequence a set of vital genes from patients. Consequently, certain lncRNAs have been identified in diverse malignant tumors, serving as reliable indicators of prognosis as well as treatment responsiveness^24,25^.

Several studies have highlighted the significance of lncRNAs in BC^26,27^, revealing their vital role in metabolism^28^. They are identified to be closely involved with metabolic processes in cancer^29^, potentially influencing glycolysis activity and cell proliferation by altering metabolism-related signaling pathways^30^. Thus, it becomes essential to identify a metabolism-related lncRNA prognostic signature in BC patients.

The novelty and technicality of this research were evident in the prognostic signature, which comprises nine metabolism-related lncRNAs (SIRLNT, SIAH2-AS1, MIR205HG, USP30-AS1, MIR200CHG, TFAP2A-AS1, AP005131.2, AL031316.1, C6orf99). This signature distinguished patients at different risk levels and had been identified as a significant independent factor for patients with BC. The ROC curve suggested that the newly discovered metabolism-related lncRNA signature exhibited a moderate predictive performance for OS. A newly proposed nomogram was expected to guide doctors in making informed treatment decisions. Our investigation revealed that immunotherapy and targeted therapy demonstrated potential effectiveness for BC patients across diverse risk groups. Advancements in the understanding of metabolism-related lncRNAs could facilitate the development of a more comprehensive mechanistic insight into BC, thus catalyzing transformative progress in clinical practice.

Among the identified lncRNAs, MIR205HG, USP30-AS1, MIR200CHG, and TFAP2A-AS1 were associated with tumor progression, each mediating different processes of tumor development. LncRNA miR205HG interacts with HNRNPA0 mRNA and then inhibits the migration and invasion of esophageal carcinoma cells^31^. Repression of mitophagy by USP30-AS1 may have a role in the development of glioma tumors^32^. MIR200CHG promotes breast cancer proliferation, invasion, and treatment resistance^33^. In gastric cancer cells, TFAP2A-AS1 was confirmed to inhibit the proliferation and migration^34^. SIAH2-AS1, AP005131.2, AL031316.1, and C6orf99 parts of lncRNAs risk models were implicated with autophagy, immunity, and hypoxia, suggesting a close association of tumor metabolism with these processes. In recent years, numerous studies, including those related to miRNA–lncRNA interaction prediction, have been conducted in the field of bioinformatics^35,36^. In the Sankey diagram, we identified several lncRNAs that were associated with key genes, including GBE1, HK3, PGM1, PYGL, and UGP2, which were involved in glycogenesis. Fluctuations in specific metabolite levels can contribute to the development of cancer. Detecting such deviations in metabolite levels can assist in disease diagnosis^37^.

Moreover, low-risk patients showcased higher numbers of B cells, CD8^+^ T cells, T helpers, and TIL cells, stressing the importance of metabolism-related lncRNAs in controlling tumor immune infiltration. It was observed that immune infiltration in BC was linked to these lncRNAs^38,39^. With the tumor environment related to the outcome of immune checkpoint inhibitor treatments^40,41^, our study found that low-risk patients demonstrated higher levels of CTLA-4, PD-1, and PD-L1, suggesting that immunotherapies targeting these entities could be more beneficial for such patients. This casts light on tumor immunotherapy in a novel way. When coupled with endocrine therapy in advanced BC, CDK4/6 inhibitors have been demonstrated to improve response rates and prolong disease control^42,43^. The antitumor efficacy of small compounds was determined through in vitro testing^44^. Several researchers have developed novel deep learning predictive models to identify and avoid serious cardiotoxicity inhibitors^45^. Interestingly, CDK4, CDK6, and PIK3CA were significantly overexpressed in the high-risk group, implying that CDK4/6 inhibitors and PIK3CA inhibitors may improve outcomes in the high-risk group.

We performed molecular verification using BC cell line MCF-7, which revealed high expression levels of C6orf99. BC cells displayed augmented proliferation, migration, and invasion influenced by C6orf99, thereby proposing C6orf99 as a potential oncogene in BC, contributing to cancer proliferation and metastasis.

Single-cell multimodal sequencing techniques have become available to enhance our understanding of cancer cellular function and heterogeneity of individual cancer cells^46^. Several single-cell multimodal analysis frameworks have been developed, providing a more comprehensive understanding of cellular heterogeneity and facilitating research in biomedical diseases^47,48^. Hence, there is a necessity to advance data analysis frameworks founded on deep learning to enhance the effectiveness of data analysis.

However, our study also recognizes potential limitations of the metabolism-related lncRNAs prognostic signature that may restrict its applicability, necessitating further improvement. Although we used data from the TCGA and GEO public databases, obtaining prospective, multicenter, real-world data can substantiate our predictive model. Theoretical modeling studies of gene/protein signaling networks are crucial for understanding regulatory mechanisms and identifying potential therapeutic targets for diseases^49–51^. Future studies can be strengthened by the incorporation of more state-of-the-art computational models and technologies.

## Conclusion

In conclusion, we discovered a new metabolism-related predictive risk model in breast cancer made up of 9 lncRNAs (SIRLNT, SIAH2-AS1, MIR205HG, USP30-AS1, MIR200CHG, TFAP2A-AS1, AP005131.2, AL031316.1, C6orf99). If the nine metabolism-related lncRNA signature is verified prospectively, it has the potential to improve prediction accuracy and lead to personalized treatment for breast cancer patients.

### Supplementary Information

Supplementary Table S1.

## Data Availability

The datasets are available for download in the TCGA: https://portal.gdc.cancer.gov/ and GEO database, https://www.ncbi.nlm.nih.gov/geo/.

## Abbreviations

lncRNA: Long non-coding RNA
BC: Breast cancer
TCGA: The Cancer Genome Atlas
GEO: Gene Expression Omnibus
KEGG: Kyoto Encyclopedia of Genes and Genomes
OS: Overall survival
ssGSEA: Single sample gene set enrichment analysis
AUC: Area under the curve
ROC: Receiver operating characteristics
C-index: Concordance index
PCA: Principal component analysis
TIMER: Tumour Immune Estimation Resource
GO: Gene ontology
LASSO: Least absolute shrinkage and selection operator
CI: Confidence interval
IC50: Half maximal inhibitory concentration
