# Supplementary material for: The metabolism-related lncRNA signature predicts the prognosis of breast cancer patients
Source: Sci Rep. 2024 Feb 12;14:3500. doi: 10.1038/s41598-024-53716-7 (PMC10861477; doi:10.1038/s41598-024-53716-7)
Supplement: Supplementary file 1 — Supplementary Table S1. [file 41598_2024_53716_MOESM1_ESM.docx]

Table S1. Primers and siRNA sequences

| Name | Sequence 5'-3' |
| --- | --- |
| C6orf99-forward | TCAACGGTTCCCTCTTCGGA |
| C6orf99-reverse | CGTTCCTGTAGGACAAGGGG |
| GAPDH-forward | ACAACTTTGGTATCGTGGAAGG |
| GAPDH -reverse | GCCATCACGCCACAGTTTC |
| C6orf99-siRNA #1-forward | AGUAACACCAGAACUGUUCAC |
| C6orf99-siRNA #1-reverse | GAACAGUUCUGGUGUUACUCA |
| C6orf99-siRNA #2-forward | UUGUACUUAAAUUUACCACGG |
| C6orf99-siRNA #2-reverse | GUGGUAAAUUUAAGUACAAAA |
